# Surgical Management of a Rare Case of Congenital DoubleUpper Lip

**DOI:** 10.1155/2011/824634

**Published:** 2011-12-08

**Authors:** Adit Srivastava, Ajit Parihar, Romesh Soni, M. C. Shashikanth, T. P. Chaturvedi

**Affiliations:** ^1^Faculty of Dental Sciences, Institute of Medical Sciences, Banaras Hindu University, Varanasi 221005, India; ^2^Department of Oral Medicine and Radiology, UP Dental College, Lucknow 227105, India

## Abstract

Congenital double lip is a rare developmental anomaly which usually involves the upper lip. It may occur in isolation or as a part of Ascher's syndrome. The occurrence of double lip may result in facial deformity especially when patient attempts to talk, smile, or even try to show the teeth. It affects esthetics and also interferes with speech and mastication. Although surgery may be undertaken to facilitate speech and mastication, majority of cases are operated for cosmetic reasons. A case of congenital double upper lip which was surgically treated for cosmetic reason is reported.

## 1. Introduction

Double lip is one of the rarest forms of lip abnormality [1]. The term double lip is used to describe deformity of either upper or lower lip in which a fold of labial mucosa is apparent at rest or at smiling. It affects upper lip more commonly than lower lip and rarely both lips are affected [2]. Double lip usually manifests as two masses of hyperplastic tissue on either side of midline which can sometimes be asymmetrical [3]. Double lip can either be congenital or acquired. Acquired deformity may be secondary to trauma or habits such as sucking lips between diastema or ill fitting dentures. The congenital double lip is believed to be present at birth and becomes more prominent after eruption of teeth. It may occur in isolation or as part of Ascher's syndrome [1, 4]. There appears to be male gender predilection of 7 : 1 for this anomaly [3]. The treatment is surgical and usually indicated for cosmetic reasons [5]. Recurrence is rare. A case of double lip is reported in a 35-year male which was treated by excision of excess of mucosal and submucosal tissues.

## 2. Case Report

**Figure 1:**
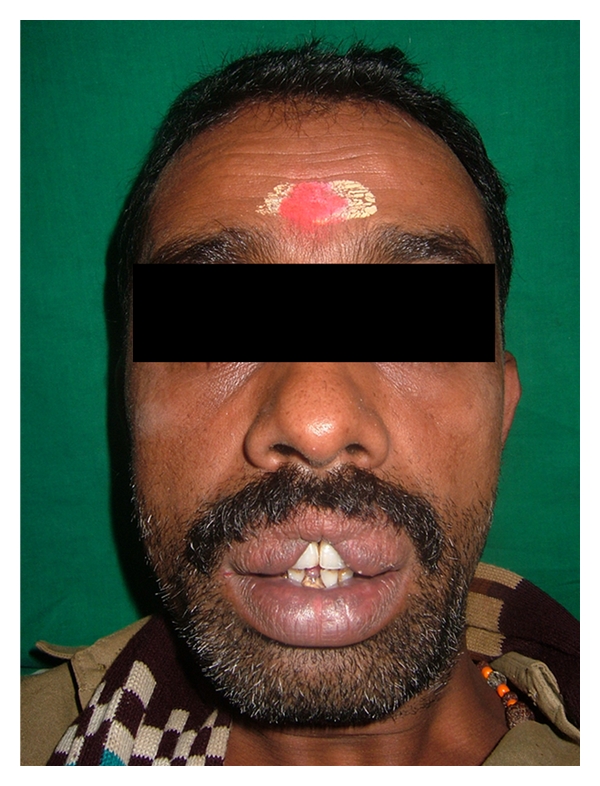
Pre-operative patient's photograph.

A 35-year-old male patient came to our department with chief complain of deposits on his teeth and also complained that his upper lip was large and unusual because of which he felt ashamed and wanted to get it corrected (Figure 1). There was no family history of double lip and no previous history of trauma or surgery.

**Figure 2:**
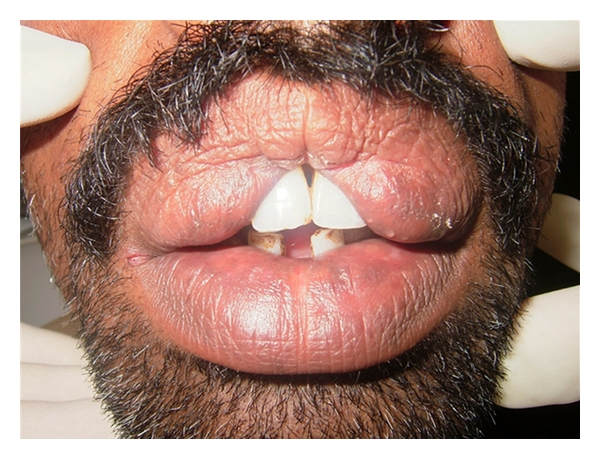
Frontal view of double lip.

**Figure 3:**
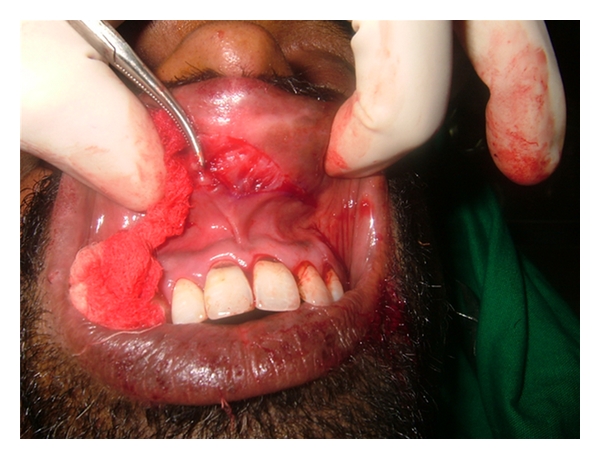
Dissection of hyperplastic upper labial tissues.

**Figure 4:**
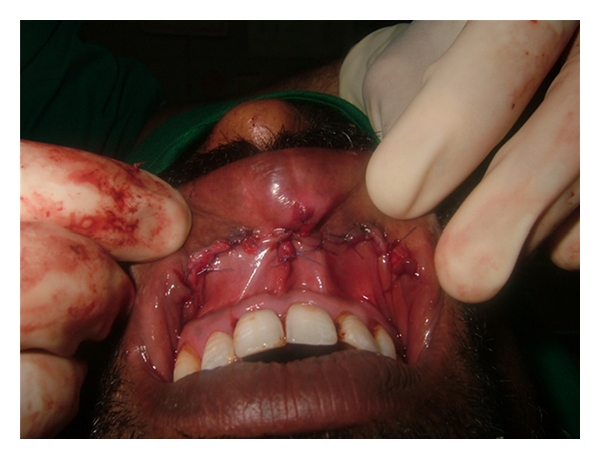
Sutures placed.

**Figure 5:**
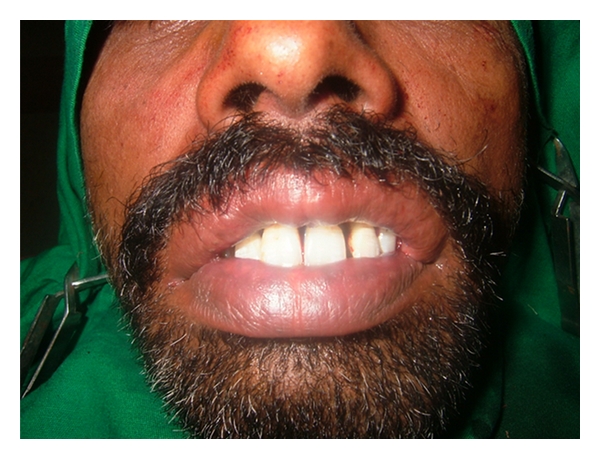
Immediate postoperative view.

**Figure 6:**
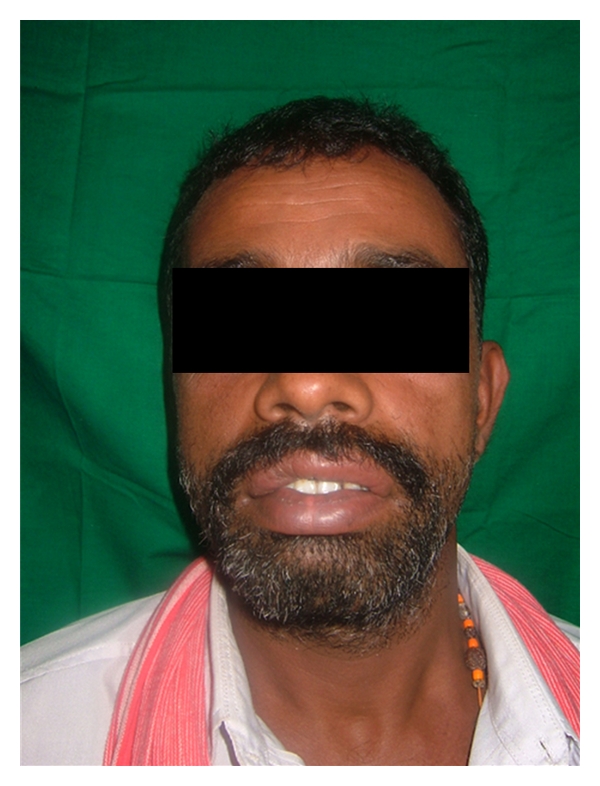
Postoperative view after 1 week.

Upon examination, a thick upper lip was seen with midline constriction band between two mucosal bulges which was even visible at rest (Figure 2). There was no blepharochalasis and no thyroid gland enlargement. A provisional diagnosis of congenital double lip was made and under differential diagnosis chelitis glandularis, angioedema, vascular tumors, chelitis granulomatosa, mucocele, salivary gland tumor, and inflammatory fibrous hyperplasia were considered. His general blood picture was within normal limits. Under local anesthesia, hyperplastic upper labial tissue was demarcated and excised by a transverse elliptical incision from one commissure to another with central Z plasty using blunt and sharp dissection (Figure 3). The minor salivary glands in the field were also removed. The surgical defect was closed using interrupted vicryl sutures (Figure 4). A light pressure dressing was placed over upper lip for first 24 hours. Histopathological report of the specimen showed normal labial mucosa with mucosal glands and capillaries. Sutures were removed after a week and postoperative healing was uneventful (Figures 5 and 6). Patient was satisfied with his appearance and new look. Patient was followed up to 2 years with no recurrence, after which was lost for follow-up.

## 3. Discussion

Double lip is an uncommon congenital or acquired anomaly that can have important consequences for the patient [3]. It is generally reported that double upper lip is not evident when lips is at rest but excess fold of tissue projects beyond the vermilion border when lip is tense as in smiling or laughing position [2, 6]. However in our case, hyperplastic tissue was visible even at rest and became accentuated when he tried to show his teeth. Double upper lip may be present with central constriction due to attachment of upper labial frenum which was similar to our case but cases without central constriction have also been reported [7]. Double lip has generally been associated with Ascher's syndrome but Parmar and Muranjan [8] and Costa-Hanemann et al. [9] described its presences with cases having different clinical presentation and suggested that it was a new syndrome.

The treatment of double lip is surgical mostly done for cosmetic reasons. Sometimes surgery may be necessary when hyperplastic tissue interferes with speech and mastication. The surgery involves excision of excess mucosa and submucosal without involving underlying muscular layer. Treatment can either be done under general or local anesthesia. Due to presence of central constriction we used double elliptical incisions combined with central vertical Z plasty [7]. However W plasty can also achieve similar results [10]. Surgical experience of the surgeon should dictate this choice [3]. Because of progressive nature of disorder or suspected Ascher's syndrome the patient should be followed up because blepharochalasis and nontoxic enlargement of thyroid gland can develop later [5]. Although recurrence is rare, Palma and Taub [3] reported a case of upper lip with recurrence. The patient in this report was followed up for 2 years before he stopped attending. No recurrence was seen during this period.
